# P-1215. In vitro activity of Sulbactam-durlobactam and Standard-of-Care Antibiotics against Acinetobacter baumannii-calcoaceticus complex isolates from wound and urinary tract sources: ACNBio Study 2023-2025

**DOI:** 10.1093/ofid/ofaf695.1408

**Published:** 2026-01-11

**Authors:** Tomefa E Asempa, Elizabeth Cyr, Jill Argotsinger, Eric T Beck, Robin R Chamberland, Anne R Daniels, Rachel Liesman, Philip Gialanella, Jonathan Hand, Amanda Harrington, Romney Humphries, Holly Huse, Robert Hamilton-Seth, Wesley D Kufel, Scott W Riddell, Lars Westblade, Jamie Marino, Navaneeth Narayanan, Thomas Kirn, Virginia M Pierce, Raghava Potula, Tsigereda Tekle, Patricia J Simner, Brian Mochon, Julia Hankins, Mark Fisher, Rebekah Dumm, Manohar B Mutnal, Timothy C Jenkins, Violeta Chavez, Robert Tibbetts, Andrew E Clark, Christine A Vu, Lilian M Abbo, Octavio Martinez, David P Nicolau

**Affiliations:** Hartford Hospital, Hartford, CT; Hartford Hospital, Hartford, CT; Advocate Lutheran General Hospital, Park Ridge, IL; Department of Microbiology, ACL Laboratories, West Allis, Wisconsin; Saint Louis University School of Medicine, St. Louis, Missouri; Froedtert and Medical College of Wisconsin, Milwaukee, Wisconsin; Medical College of Wisconsin, Milwaukee, Wisconsin; Montefiore Medical Center, Albert Einstein College of Medicine, Bronx, New York; Ochsner Health, New Orleans, LA; Loyola University Chicago, Maywood, IL; Vanderbilt University Medical Center, Nashville, Tennessee; Harbor-UCLA Medical Center, Torrance, California; University of Kansas Health System, Kansas City, Kansas; Binghamton University School of Pharmacy Sciences, Binghamton, NY; State University of New York Upstate Medical University, Syracuse, New York; Weill Cornell Medicine, New York, New York; Weill Cornell Medicine, New York, New York; Rutgers University Ernest Mario School of Pharmacy & Robert Wood Johnson University Hospital, New Brunswick, NJ; Rutgers Robert Wood Johnson Medical School, New Brunswick, New Jersey; University of Michigan Medical School, Ann Arbor, Michigan; Temple University Health System, Philadelphia, Pennsylvania; Johns Hopkins, Baltimore, Maryland; Johns Hopkins School of Medicine, Baltimore, MD; Phoenix Children's Hospital, Phoenix, Arizona; University of Kansas Health System, Kansas City, Kansas; ARUP Laboratories; University of Utah Health, Salt Lake City, Utah; Washington University School of Medicine; Baylor Scott and White, Temple, Texas; Denver Health, Denver, Colorado; University of Texas, Houston, TX, United States, Houston, TX; Henry Ford Health, Detroit, Michigan; University of Texas Southwestern Medical Center, Dallas, Texas; Jackson Memorial Hospital, Miami, FL; University of Miami School of Medicine, Miami, Florida; Jackson Health System/University of Miami, Miami, FL; Hartford Hospital, Hartford, CT

## Abstract

**Background:**

*Acinetobacter baumannii* most often causes nosocomial bacteremia and pneumonia. However, *A. baumannii* is becoming an important cause of a broader range of infections including skin and soft tissue infection (SSTI) and urinary tract infections (UTI). This study aims to evaluate the *in vitro* activity of sulbactam-durlobactam and other clinically utilized antibiotics against *A. baumannii-calcoaceticus* complex isolates isolated from non-respiratory and non-bloodstream sources.

**Figure:**
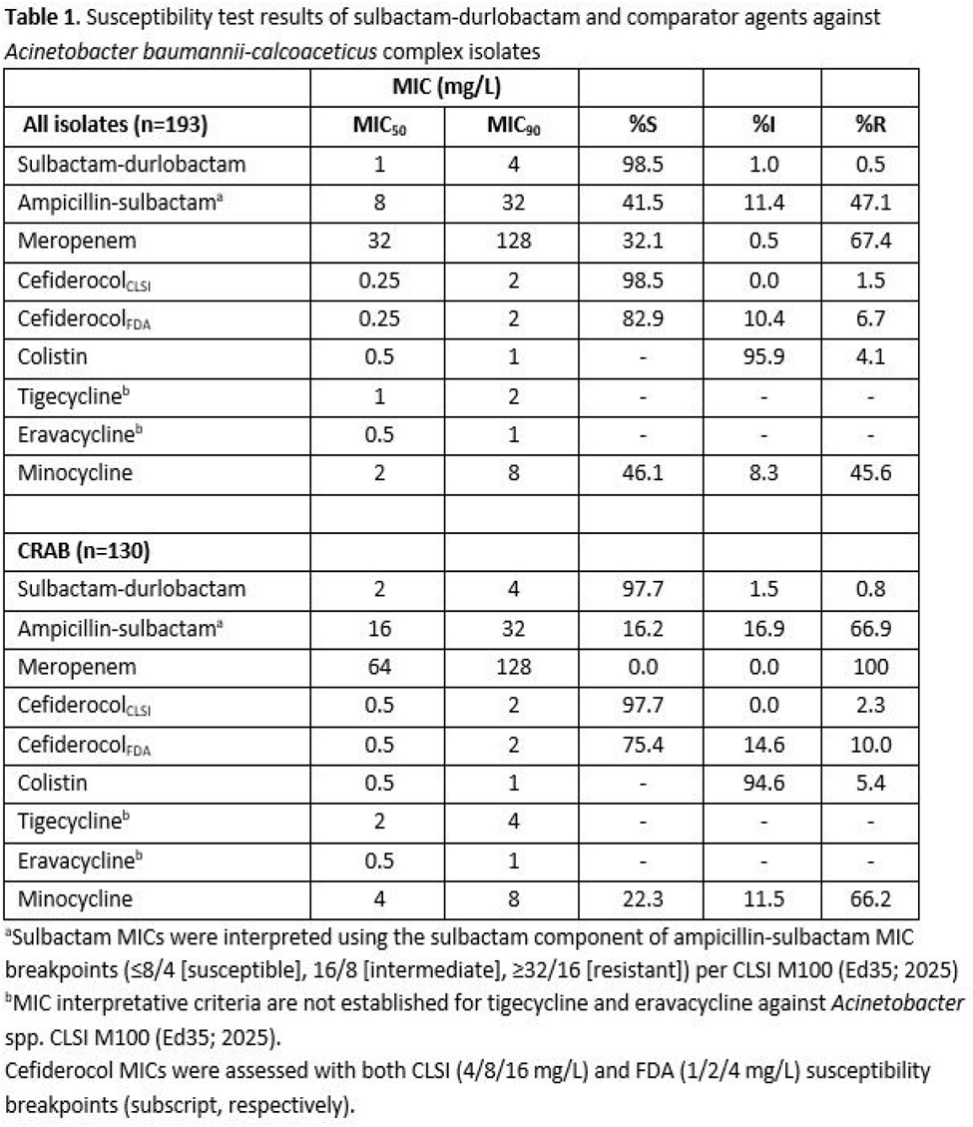

**Methods:**

Samples included 193 *A. baumannii-calcoaceticus* complex isolates collected from 2023-2025 across 19 states. Susceptibility tests for sulbactam-durlobactam (durlobactam fixed concentration of 4 mg/L), and comparator agents were conducted by manual broth microdilution and interpreted according to CLSI and FDA (cefiderocol) standards.

**Results:**

*A. baumannii* complex isolates were primarily cultured from skin and soft tissue (56.5%), followed by urinary tract (31.6%) and other sources (11.9%) including bone biopsy, fluid aspirate etc. ICU patients contributed 20% of the isolates. Sulbactam-durlobactam was observed to be highly active (98.5% susceptible [S]; MIC_50/90_ 1/4 mg/L), demonstrating greater activity than ampicillin-sulbactam (41.5% S; MIC_50/90_ 8/32 mg/L) and meropenem (32.1% S; MIC_50/90_ 32/128 mg/L). Sulbactam-durlobactam also displayed high susceptibility rates across sample sources, ranging from 97.3% (skin and soft tissue) to 100% (urine). The MIC_50/90_ and susceptibility rates for all other agents including tetracycline derivatives are shown in Table 1. Notably, among the carbapenem-resistant *A. baumannii* (CRAB) isolates (n=130) in the collection, sulbactam-durlobactam retained high activity (97.7% S) relative to ampicillin-sulbactam (16.2% S). Cefiderocol inhibited 97.7% and 75.4% of isolates at CLSI and FDA susceptible breakpoints, respectively.

**Conclusion:**

The data reported here are consistent with results from surveillance studies among non-respiratory and bloodstream isolates and show that sulbactam-durlobactam demonstrates *in vitro* activity against clinical *A. baumannii* complex isolates from urinary tract and SSTI sources, including isolates that are resistant to ampicillin-sulbactam, carbapenems, and cefiderocol.

**Disclosures:**

Tomefa E. Asempa, PharmD, Innoviva: Grant/Research Support Robin R. Chamberland, PhD D(ABMM), bioMerieux: Advisor/Consultant|Pattern Bioscience, Inc.: Advisor/Consultant|Pattern Bioscience, Inc.: Grant/Research Support Jonathan Hand, MD, AstraZeneca: Advisor/Consultant|AstraZeneca: Grant/Research Support|Ferring: Grant/Research Support|Innoviva: Advisor/Consultant|Janssen: Grant/Research Support|Pfizer: Advisor/Consultant|Pfizer: Grant/Research Support|Scynexis: Grant/Research Support|The Antibiotic Resistance Leadership Group (ARLG): Grant/Research Support|The Antibiotic Resistance Leadership Group (ARLG): Honoraria Amanda Harrington, PhD, Beckman Coulter: Grant/Research Support|bioMerieux/BioFire: Advisor/Consultant|bioMerieux/BioFire: Grant/Research Support|bioMerieux/BioFire: Honoraria|BioRad: Advisor/Consultant|Selux: Grant/Research Support Wesley D. Kufel, Pharm.D., BCPS, BCIDP, Merck & Co.: Grant/Research Support|Shionogi, Inc: Grant/Research Support|Shionogi, Inc: Honoraria Lars Westblade, PhD, Elements Materials Technology: Grant/Research Support|Hardy Diagnostics: Grant/Research Support|Melinta Therapeutics: Grant/Research Support|Selux Diagnostics: Grant/Research Support|Shionogi: Advisor/Consultant|SNIPRBIOME: Grant/Research Support Thomas Kirn, MD PhD, BD: Advisor/Consultant|BD: Honoraria Brian Mochon, PhD, D(ABMM), Shionogi: Advisor/Consultant Mark Fisher, PhD, Shionogi Inc.: Advisor/Consultant Rebekah Dumm, PhD D(ABMM), BD: Advisor/Consultant|BD: Grant/Research Support|Biomerieux: Advisor/Consultant|Biomerieux: Grant/Research Support|Diasorin: Grant/Research Support|Pattern Biosciences: Grant/Research Support|Qiagen: Grant/Research Support|Roche Diagnostics: Advisor/Consultant|Shionogi: Advisor/Consultant David P. Nicolau, PharmD, CARB-X: Grant/Research Support|Innoviva: Advisor/Consultant|Innoviva: Grant/Research Support|Shionogi: Advisor/Consultant|Shionogi: Grant/Research Support

